# Draft Genome Sequence of the Aerobic Anoxygenic Phototrophic Bacterium *Roseobacter* sp. Strain OBYS 0001, Isolated from Coastal Seawater in Otsuchi Bay, Japan

**DOI:** 10.1128/MRA.00488-21

**Published:** 2021-07-15

**Authors:** Yuki Sato-Takabe, Yu Nakajima, Yuya Tsukamoto, Koji Hamasaki, Takuhei Shiozaki

**Affiliations:** a Atmosphere and Ocean Research Institute, The University of Tokyo, Kashiwa, Chiba, Japan; b Institute for Extra-Cutting-Edge Science and Technology Avant-Garde Research (X-star), Japan Agency for Marine-Earth Science and Technology, Yokosuka, Kanagawa, Japan; University of Southern California

## Abstract

Here, we report the draft genome sequence of the aerobic anoxygenic phototrophic bacterium *Roseobacter* sp. strain OBYS 0001, isolated from coastal seawater in Ostuchi Bay, Japan. This genome sequence could be useful for our understanding of the variation in photosynthesis-related genes among aerobic anoxygenic phototrophs.

## ANNOUNCEMENT

Aerobic anoxygenic phototrophic bacteria (AAnPB) are photoheterotrophic organisms containing bacteriochlorophyll (BChl) *a* (1). *Roseobacter* sp. strain OBYS 0001 is a Gram-negative AAnPB belonging to the phylum *Proteobacteria*. The genus *Roseobacter* is one of the genera in which aerobic BChl *a*-containing bacteria were first discovered (2). Strain OBYS 0001 was isolated from coastal seawater in Otsuchi Bay, Japan (3). The taxonomic status of the isolate was established by Sato-Takabe et al. (3). This strain shows effective green-light-capturing ability (3), which can increase photosynthetic competence under organic substrate-deficient conditions (4).

Genomic DNA from strain OBYS 0001, grown in ZoBell 2216E liquid medium at 30°C for 14 days under dark conditions, was extracted using phenol-chloroform and ethanol precipitation (5). The MGIEasy FS PCR-free DNA library prep set (MGI) was used for library preparation, and paired-end sequences (150 bp from each end) were obtained using the DNBSEQ instrument (MGI). Genome assembly was performed using SPAdes 3.15.2, with slight modifications (6). The assembled sequences were annotated using the DDBJ Fast Annotation and Submission Tool (DFAST; https://dfast.nig.ac.jp) and Prokka, version 1.14.6 (7). Default parameters were used for all software unless otherwise specified. 16S rRNA gene sequence taxonomic identification using EzBioCloud (8) confirmed that strain OBYS 0001 is most closely related to Roseobacter litoralis Och 149 (100% identity). The genome sequence of *Roseobacter* sp. strain OBYS 0001 consists of 42 scaffolds using SPAdes (total length, 4,710,781 bp; *N*_50_, 415,052 bp). The number of reads generated for the genome was 1.629 million (5.43 Gbp). The genome size was estimated using DFAST, and the genome coverage value was 347×. The Q30 score for the genome sequencing for strain OBYS 0001 was 80.8%. The estimated genome size of the strain was similar to those of sequenced strains belonging to the genus *Roseobacter* (e.g., 4.51 Mbp for Roseobacter litoralis OCH 149 [GenBank accession number CP002623.1], 4.13 Mbp for Roseobacter denitrificans Och114 [CP027407.1], 3.86 Mbp for Roseobacter ponti MM-7 [CP048789.1], and 4.47 Mbp for Roseobacter cerasinus AI77 [NZ_BLIV00000000.1]), and the GC content for OBYS 0001 (57.3%) was similar to those of the *Roseobacter* strains (*R. litoralis*, 57.2%; *R. denitrificans*, 59.0%; *R. ponti*, 60.5%; *R. cerasinus*, 61.0%).

For *Roseobacter* sp. strain OBYS 0001, DFAST and Prokka identified 4,558 protein-coding sequences (CDSs) and 4,533 CDSs, respectively. Both tools also identified 41 tRNA and 3 noncoding rRNA genes for OBYS 0001.

Because the photosynthetic characteristics for strain OBYS 0001 have already been reported (3, 4), the present genomic data will be helpful for a more comprehensive understanding of aerobic anoxygenic phototrophs in the future.

### Data availability.

The whole-genome shotgun project for *Roseobacter* sp. strain OBYS 0001 has been deposited in DDBJ/EMBL/GenBank under the accession numbers BPES00000000.1 for the assembly and DRR287723 and DRA011876 for the raw reads.
